# Synthesis and characterization of nanozeolite based composite fertilizer for sustainable release and use efficiency of nutrients

**DOI:** 10.1016/j.heliyon.2021.e06091

**Published:** 2021-01-31

**Authors:** M.Z.H. Khan, M.R. Islam, N. Nahar, M.R. Al-Mamun, M.A.S. Khan, M.A. Matin

**Affiliations:** a Dept. of Chemical Engineering, Jashore University of Science and Technology, Jashore 7408, Bangladesh; b Environmental Laboratory, Arsenic Center, Asia Arsenic Network, Jashore 7400, Bangladesh; c Dept. of Glass and Ceramic Engineering, Bangladesh University of Engineering and Technology, Dhaka 1000, Bangladesh

**Keywords:** Nanozeolite, Nanoparticles, Micro-nutrients, Nano-fertilizer, Sustainable release

## Abstract

In this research work, we propose macronutrients incorporated slow-release based nano-fertilizer using nanozeolite as a carrier. A simple chemical approach was used to synthesis the proposed nanozeolite composite fertilizer (NZCF). To gain an insight into the properties, morphology and structure of the synthesized NZCF, it was further characterized by different techniques such as powder XRD, FT-IR, SEM, and TG/DTA. A considerable enhancement of the quality and the water retention capacity of the soil was observed as a result of applying the proposed NZCF when compared with a commercial fertilizer. Furthermore, the swelling ratio and the equilibrium water content of NZCF were compared to the commercial fertilizer and their effect on plant growth was observed. Slow-release studies were carried out for both NZCF and the commercial fertilizer. The results of these studies reveled that NZCF possessed a long-term release pattern of the macronutrients and that showed a great potential for promoting plant growth. Hence, the prepared nanocomposite fertilizer can be safely used as an environment-friendly source of nutrients to enhance plant growth.

## 1 Introduction

Currently, conventional fertilizers used in agriculture to enhance the crops production is a common trade worldwide. However, the large-scale use of commercial fertilizers decreases the efficiency of utilizing the soil nutrients [1, 2]. Heavy metals may enter into the soil, plant system and food chain due to excess application of fertilizer and that is a great threat to lives [3]. The total demand for nitrogen worldwide is calculated to be 112.9 million tons in 2015 and about 60–75% of nitrogen is utilized in the fertilizers industry [4]. Therefore, the extended presence of commercial fertilizer pollutes both underground and surface water causing nitrate contamination and eutrophication. The toxic chemicals that are releasing from fertilizers by water runoff eventually reach water bodies such as oceans, river, ponds and that causes great damage to the ecosystem. The use of conventional fertilizer creates huge wastes that directly or indirectly cause various health concerns and has negative impacts on economy.

It's a great challenge to produce sufficient crops with these limited lands for the overpopulated world without damaging soil nutrients. During the last decades, nanotechnology created an industrial revolution due to the unique properties of nanomaterials. Their noble characteristics were well utilized in the controlled delivery of pesticides, fertilizers, nutrients and genetic materials. Furthermore, it was reported that nanomaterials were used for holding plants essential nutrients for a long time [5, 6, 7]. Nanofertilizer refers to nano-sized fertilizer that contains nanoparticles and encapsulation of nutrients, and can systematically release micro and macronutrients to target specific sites in plants. The nanostructured elements in nanofertilizer is often incorporated in a carrier complex by absorption or adsorption in a matrix. Chitosan, polyacrylic acid, clay and zeolite were previously reported as carriers for nanofertilizers [2, 8, 9]. Nanoporous zeolite attracts the attention in farming to increase the fertilizer use efficiency of crops over the adverse effect of chemical fertilizer on the agriculture ecosystems. Due to the high surface area, mesoporous structure and high nutrients loading capacity of nonporous zeolite, it was previously reported that its slow-release of nanocarriers was utilized to enhance the nutrient retention capacity of soil [10, 11].

Generally, macronutrient is used to increase soil fertility which in turn promotes plant growth [12]. The addition of macronutrients to farming land is essential to compensate for the lack of minerals and nutrients contents. The primary and secondary macronutrients such as potassium, nitrogen, phosphorus, silicon, calcium are essential for plant gardening and cultivation. To improve the nutrients use efficiency and to prevent the loss of nutrients to the environment, nanofertilizers and nanocomposites were widely used as a slow-release fertilizer in farming [13]. The incorporation of various macro and micronutrients in zeolite effectively reduced nutrients deficiencies of soil as reported by many researchers [12, 13, 14, 15, 16]. Although most of the previous reports focused on nanozeolite fertilizers incorporated with different nanoparticles, the effect of nanozeolite hybrid fertilizer on slow-release was never studied.

This work presents the synthesis and characterization of nanozeolite based fertilizer-impregnated with macronutrients. Nutrient uptake capacities and slow-release study of the proposed composite fertilizer were carried out. Furthermore, a comparative study of the effect of both the prepared NZCF and a commercial fertilizer on the growth of lettuce plantation was undertaken.

## 2 Materials & method

### 2.1 Chemicals and reagents

All the chemicals used in the experiment are of analytical grade and used without further purification. Deionized (DI)water was used for all sample preparations.

### 2.2 Synthesis of nanozeolite

Nanozeolite was prepared by using a simple co-precipitation method as was previously described by Bansiwal *et al.* [17]. At first, sodium silicate solution (73.3g/100ml of distilled water) and 8.33ml of ethylene glycol were mixed in a three-necked round bottom flask. Further, the flask was fitted with a reflux condenser and dropping funnels. Then, the mixture was stirred for 30 min with a magnetic stirrer at a temperature of 50–60 °C. Next, a pre-prepared aluminum sulfate solution (26.23g/83ml) and sodium hydroxide solution (10g/83ml) were added dropwise while stirring and maintaining the same temperature. After completing the reaction, the pH level of the solution was neutralized. Later, the sample was filtered, dried at 105 °C and finally was annealed at 650 °C to obtain the desired porous grey colored zeolite.

### 2.3 Preparation of nano-composite fertilizer

NZCF was prepared by impregnating the macronutrients into nanozeolite. A 30g of nanozeolite, a 150 ml of Di water and 5% solution of macronutrients (Na, P, K, Ca, Mg and S) in the form of their salts (NaH_2_PO_4_. 2H_2_O, MgSO_4_. 7H_2_O, Ca_3_(PO_4_)_2_, KCl, and NaNO_3_) were added and the mixture was stirred for 3 hours. Finally, the solution was filtered, oven-dried at 105 °C and blended at 12000 rpm to get fine ground particles of NZCF that was then stored in an airtight container to stay dry.

### 2.4 Treatment of NZCF on plants

To test the applicability of the prepared fertilizer, lettuce plants were treated with NZCF. Furthermore, two other experiments were carried out with commercial nitrogen, phosphorus and potassium (NPK) fertilizer in order to evaluate and compare the effect of both the commercial and proposed fertilizers on plant growth, development, life span and translocation. All experiments were conducted under outdoor conditions for 6 weeks. Both experiments were carried out with the same quality compost soil and standard doses of NPK fertilizer were added according to the regulations the Ministry of Agricultural of local government.

### 2.5 Characterization of nanocomposite

To identify the presence of functional groups, Fourier transforms infrared spectra (FTIR) study was performed with a resolution of 1 cm^−1^ in the wavelength range 500–4000 cm^−1^. X-ray diffraction (XRD) measurement was carried out to investigate the structural behavior and formation of as-synthesized NZCF. Debye-Scherer equation was used to calculate the size of different nanoparticles while their surface morphology was investigated by scanning electronic microscope (SEM). TG/DTA analyses were conducted to understand the weight loss and reaction type of the synthesized NZCF.

### 2.6 Salt index (SI) analysis

1g of the prepared fertilizer was taken in a beaker and 1g of sodium nitrate (NaNO_3_) with 200ml of distilled water were taken in another beaker. The SI was measured as the ratio of conductivities of the two solutions [7]. The Eq. (1) represents the calculation of the salt index.

(1) $$\text{SI}=\frac{\text{electrical coductivitivity of }1\text{g fertilizer in water }}{\text{electrical coductivitivity of }1\text{g of NaNO}3\text{ in water}}\times 100\%$$

### 2.7 Swelling ratio (SR) and equilibrium water content (EWC) analysis

The swelling ratio is expressed as the rise in the weight of the sample after soaking in water [18]. In this study, 1g of the prepared fertilizer was taken and dipped in 200 ml of distilled water. Next, the sample was allowed to swell for 24 h at ambient temperature and pressure conditions. Finally, after filtration, the weight of the wet sample was monitored.

The SR and EWC of the fertilizer was determined by Eqs. (2) and (3) respectively.

(2) $$\text{SR}=\frac{Ws-W_{d}}{W_{d}}$$

(3) $$\text{EWC }=\frac{Ws-W_{d}}{W_{S}}\times 100\%$$

Where,

Ws = the soaked weight of fertilizer
Wd = the dry weight of fertilizer

### 2.8 Water absorption capacity (WAC) analysis

Water absorption is the percent of the water that a plant can absorb to the maximum amount of moisture for a certain period [19]. To calculate the water absorption capacity, a 1.0 g of the sample (w1) and pre-weighed petri dishes (w2) were kept in a desiccator in the wet environment for 5 days. After 5 days, the sample with the petri dishes was re-weighed (w3), kept in a desiccator and WAC was then calculated according to Eq. (4).

(4) $$\text{WAC }=\frac{W_{3}-W_{2}}{W_{1}}\times 100\%$$

### 2.9 Water retention capacity (WRC) analysis

Two pre-weighted cups, A (WA) and B (WB), were used to measure the water retention capacity (WRC). In cup A, 50.0 g of sieved soil was mixed with 30 ml of distilled water. Whereas, in cup B, 2.0 g of NZCF was mixed with 50 g of soil followed by the addition of 30 ml of distilled water. Later, both cups were reweighed (w1) after water was allowed to seep through them for 24 h. Finally, the cups were weighed daily (w2) for the following 30 days by keeping it in a glass box as previously described by Mikhak *et al.* [5]. To calculate *WRC*, the following Eq. (5) was used:

(5) $$\text{WRC }=\;\frac{W_{2}}{W_{1}}\times 100\%$$

### 2.10 Slow-release studies

Slow-release studies were performed for both water and soil to understand the leaching pattern of NZCF for 4 weeks. The experiment was conducted in a glass column using 5.0 g of NZCF, pre-analyzed soil and tap water. For slow-release study in water, 25 ml of water was collected daily and the quantity of the nutrients was determined. On the other hand, for the slow-release study of soil, 50 ml of water was collected daily from a column of soil saturated with 180 ml of pre-analyzed tap water. The nutrients release pattern was checked by analyzing collected water and soil samples.

## 3 Results

### 3.1 Characterization of NZCF

**Figure 1:**
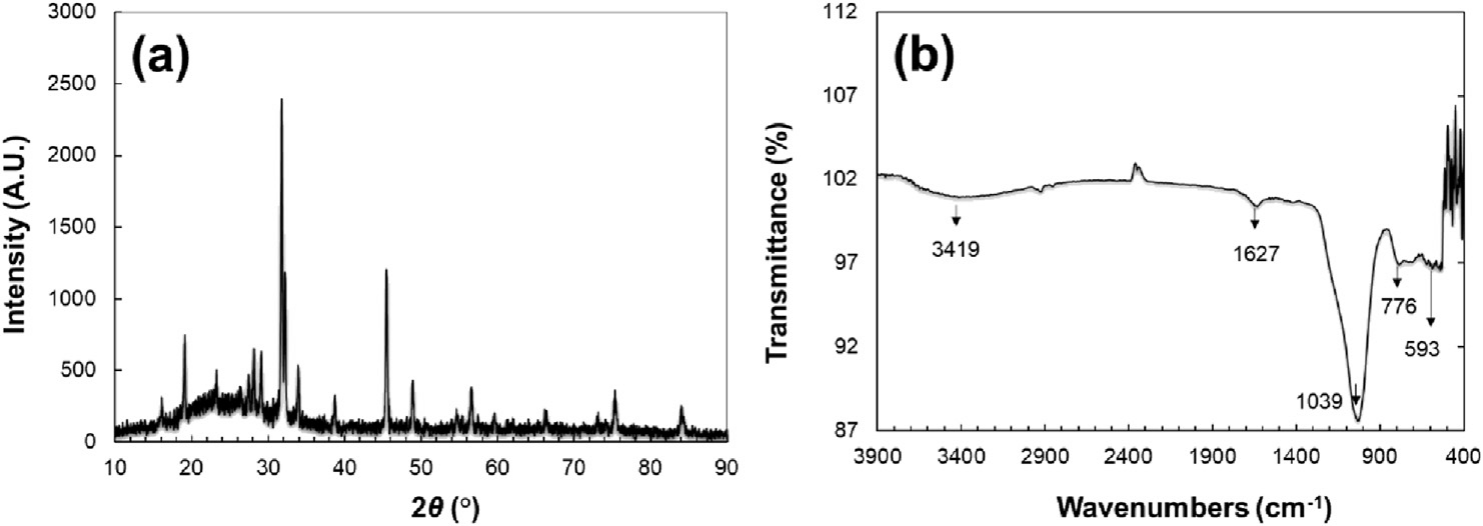
Powder XRD (a) and FTIR spectra (b) showing the chemical structure of NZCF.

Powder X-ray diffraction (XRD) analysis was performed to study the effect of different modifications on the structure stability of the modified zeolite-based NZCF and further the analysis results are presented in Figure 1a. The intensity peak observed at 2*θ* values of 23.51, 25.7, 28.2 and 31.7 are corresponding to the low crystalline structure of nanozeolite. This finding supports previous XRD analysis of nanozeolite reported by Mohanraj *et al.* [20]. The observed XRD spectrum is well-matched with the CCDC No- 01-074-1183 of sodium aluminum silicate. Moreover, new peaks appeared in the NZCF that belong to the incorporated macronutrients.

By looking at the FTIR pattern of NZCF shown in Figure 1b, one can notice the characteristic peaks at the wave numbers 1039.2 and 776.4 cm^−1^ that are assigned to the bending and stretching of Al–O and Si–O in nanozeolite structure, respectively, as was previously reported by Peter *et al.* [21]. The peaks localized at other positions in the FTIR spectra of NZCF at 1627.7 and 593.4 cm^−1^ correspond to the incorporated micronutrients in nanozeolite. The wide peak near 3400 cm^−1^ represents the bridging of OH- due to the moisture content in the porous structure of nanozeolite.

**Figure 2:**
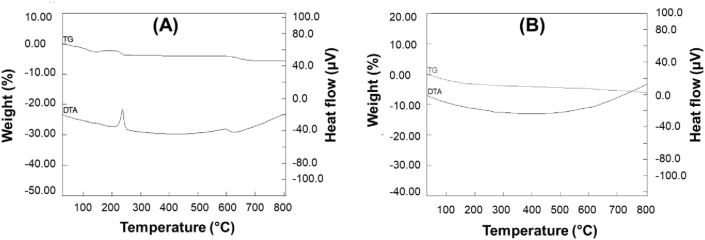
TG/DTA spectra of nanozeolite (A) and NZCF (B) showing minor weight loss.

TG and DTA analysis were conducted to study the stability and thermal degradation pattern of the prepared composites and further the analysis results are presented in Figure 2. The observed smooth weight loss throughout the study is typical for zeolites, as previously reported by other researchers [13, 17, 22]. A slight weight loss of about 3% was observed after 100 °C and that could be assigned to the loss of physically absorbed water, while almost no weight loss was observed from 310 to 630 °C as shown in Figure 2A. Moreover, two exothermic peaks were observed in the DTA curve at 300 and 600 °C for nanozeolite. However, a slow but continuous decrease in weight loss was observed for NZCF as shown in Figure 2B. This continuous decrease in weight went on until a total weight loss of approximately 9.1% and this can be described as a decomposition of the organic compounds.

**Figure 3:**
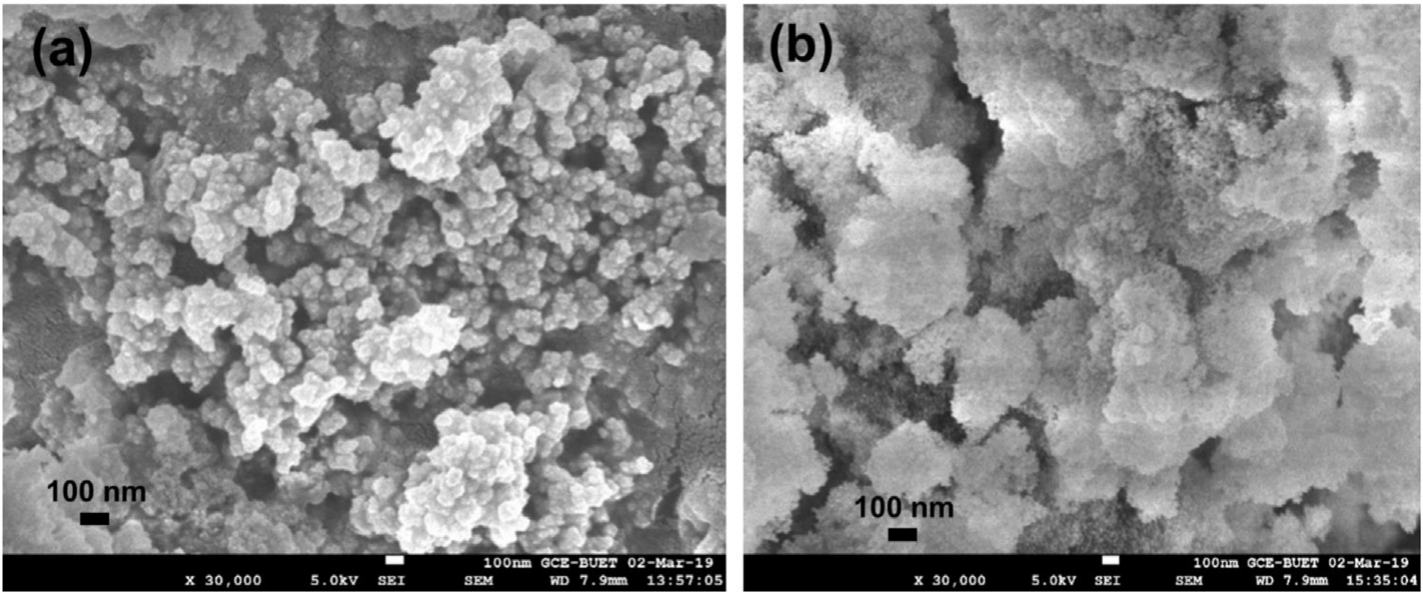
SEM images of as-prepared nanozeolite (a) and NZCF (b).

SEM measurement was performed to study the surface morphology of the as-prepared nanozeolite and NZCF, as presented in Figure 3a and b, respectively. The incorporation of macronutrients in the nanozeolite porous structure caused the spongy nature of NZCF, as seen in Figure 3b. The average size of zeolite nanoparticle was calculated from SEM images to be 40 nm.

### 3.2 Physical parameters test

To calculate the potentiality of the prepared NZCF to cause plant injury, SI measurement was conducted. The SI value was found to be approximately 11.38 and this value is much lower than that was reported for urea fertilizer by Lateef *et al.* [22]. The low value of SI indicates that the reported NZCF fertilizer is safe and suitable for the seed row placement in agriculture.

**Figure 4:**
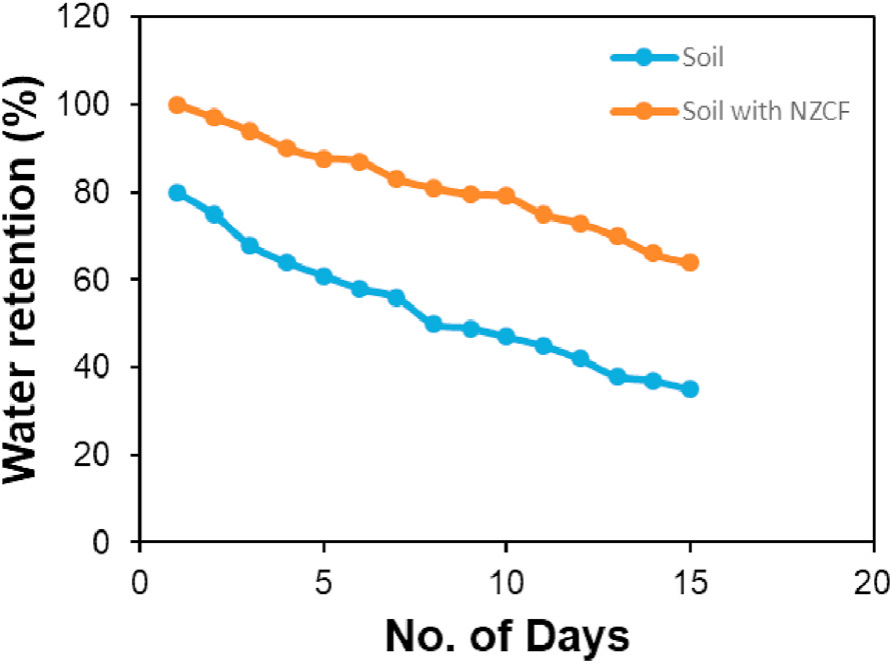
Water retention capacity of soil with and without NZCF.

Moreover, water retention (WR) value was calculated to study the water holding capacity of NZCF mixed soil. Figure 4 represents the WR value of blank soil and fertilizer mixed soil. It was observed that the WR capacity of NZCF mixed soil was 83.8, 81.5, and 66.8% on the day of 5^th^, 10^th^ and 15^th^, respectively, whereas, the value was 59.8, 47.5, and 33.2 % for blank soil on the same days. The 27% higher WR rate for NZCF mixed soil presents its higher water holding capacity and that is essential for saving water to improve the plant's health [21]. The equilibrium water content (EWC), water absorbance (WA), and swelling ratio (SR) were studied for as-prepared NZCF. The measured values for EWC, WA, and SR was 77.2, 80, and 3.3%, respectively.

### 3.3 Release of nutrients

Slow-release study was conducted both in soil and water separately to observe the release pattern of specific nutrients from the prepared fertilizer. Table 1 summarizes 14 days of nutrient release studies in soil and water after the addition of NZCF. The observed trend of nutrients release from 1-14 days indicates continuous nutrient supply to plants and that prevents leaching losses. On the contrary, Lateef *et al.* reported high leaching losses with traditional fertilizer [22]. The slow release of nutrients facilitates the growth of a healthy plant by early seed sprouting and germination.

**Table 1:** Physicochemical properties of soil and water with and without NZCF.

Parameter	Blank soil	Tap water	Soil with NZCF			Water with NZCF		
			24 h	7 days	14 days	24h	7 days	14 days
pH	8.9	7.9	8.3	7.8	7.5	7.2	7.0	7.0
Conductivity (μs/cm)	675	568	827	720	756	590	582	605
TDS (mg/l)	315	230	374	380	378	330	378	350
Ca^2+^ (mg/l)	105.8	80.6	109.0	126.6	138.3	99.2	101.3	105.3
Mg^2+^ (mg/l)	0	0	44.7	45.7	47.9	37.9	53.48	60.4
${{\text{PO}}_{4}}^{3-}$ (mg/l)	4.84	0.25	5.45	5.95	7.23	0.43	0.52	0.73
${{\text{NO}}_{2}}^{-}$ (mg/l)	0.01	0.08	0.5	1.58	1.60	0.2	0.8	1.24

### 3.4 Application on plant

**Figure 5:**
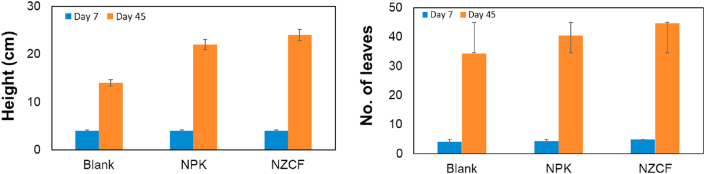
Comparison of the height (left) and number of leaves (right) of lettuce plant (*n* = 5) when blank soil, commercial fertilizer (NPK) and NZCF are used in different fertilization period.

**Figure 6:**
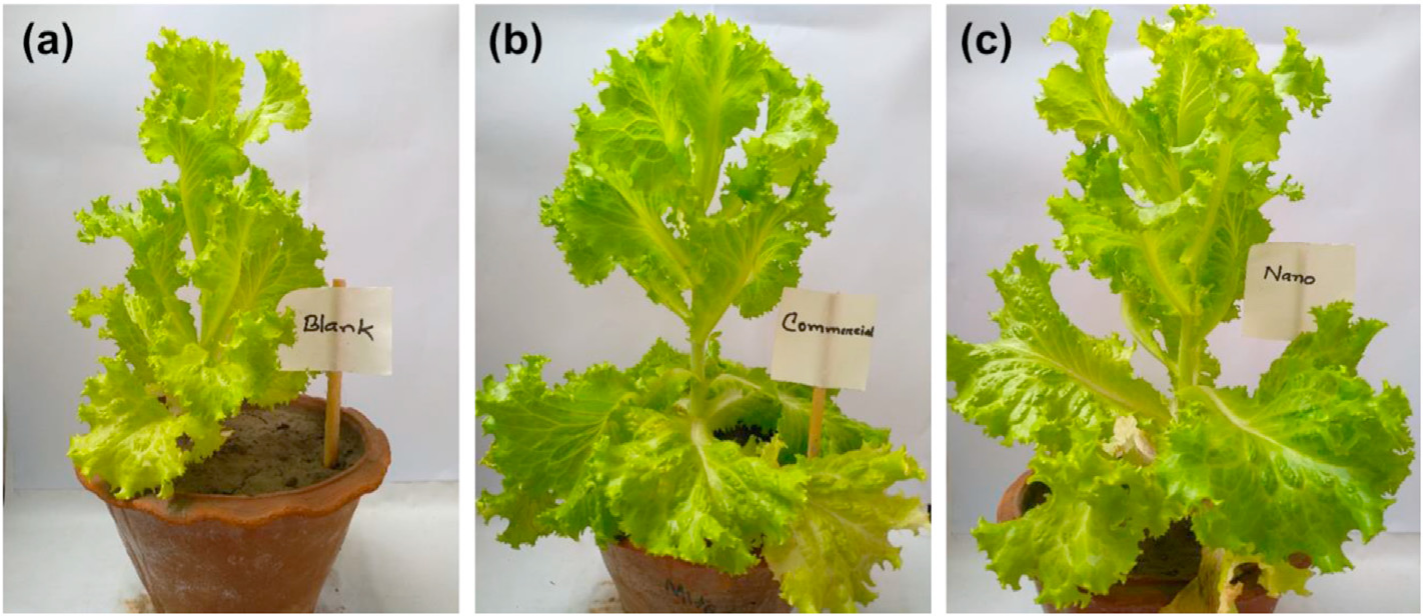
Images of lettuce plants with (a) blank soil; (b) commercial fertilizer (NPK) treatment and (c) NZCF treatment.

To study the application of the prepared NZCF on lettuce plantation, different parameters were tested. Chemical fertilizers (NPK) in doses recommended by the Local Ministry of Agriculture were added (at the rate of 1kg/42m^2^) and blank soil was used as a control. NZCF was added at the rate of 10g/42m^2^, which is 100 times less than that of the rate of commercial fertilizer. Plant height in cm and the number of branches/leaves for both the commercial fertilizer and NZCF were recorded and presented in Figure 5. The data revealed that NZCF treatment has a pivotal effect on plants. NZCF treatment significantly increased all growth parameters of lettuce plants in comparison with the blank soil (control) and plants received the recommended dose of NPK fertilizers, as shown in Figure 6.

The beneficial effects of the NZCF treatment on lettuce plants are due to the improved soil's physical, chemical and biological properties, and also the continues nutrient availability, as evidenced by the decreased soil pH, higher TDS and water retention in addition to the availability of elements to be absorbed by plant roots.

## 4 Conclusion

The experimental and analytical results suggest that nanozeolite incorporated with macronutrients led to a functional nano-fertilizer. It can be concluded that nanozeolite based composite fertilizer allowed a slow release of essential nutrients for plants growth. Water absorbance, swelling ratio, equilibrium water content, salt index and water retention studies showed good water holding capacity that can enhance soil condition without introducing any negative impacts. Furthermore, it was observed that NZCF enhanced nutrient availability in soil and further improved the soil physical, chemical and biological properties. Moreover, the proposed fertilizer showed a better plantation growth of lettuce at a lower concentration. The obtained salt index value suggests that the as-prepared NZCF nanofertilizer is safer to use compared to conventional fertilizer. Additionally, the slow-release studies of nutrients both in soil and water confirmed the long-term availability of nutrients to the plant when NZCF is applied as compared to commercial fertilizer. It can be concluded that introducing nanocomposite fertilizer in agriculture can significantly reduce the number of chemicals used while maintaining an acceptable crop yield.

## Declarations

### Author contribution statement

M. Z. H. Khan: Conceived and designed the experiments; Wrote the paper.

M. R. Islam: Performed the experiments; Wrote the paper.

N. Nahar: Performed the experiments.

M. R. Al-Mamun: Conceived and designed the experiments.

M. A. S. Khan: Analyzed and interpreted the data.

M. A. Matin: Contributed reagents, materials, analysis tools or data.

### Funding statement

This work was supported by the Ministry and Education, Government of Bangladesh (Project grant ID: PS2018774).

### Data availability statement

Data included in article/supplementary material/referenced in article.

### Declaration of interests statement

The authors declare no conflict of interest.

### Additional information

No additional information is available for this paper.
